# Supplementary material for: From sequence to enzyme mechanism using multi-label machine learning
Source: BMC Bioinformatics. 2014 May 19;15:150. doi: 10.1186/1471-2105-15-150 (PMC4229970; doi:10.1186/1471-2105-15-150)
Supplement: Additional file 2 — Java code of ml2db. Additional file ml2db_code.tar.gz contains the Java source code to run the multi-label machine learning experiments and save the results to database. The code’s Javadoc is included. [file 1471-2105-15-150-S2.zip › additional file 2/ml2db/utils/doc/uk/ac/standrews/utils/main/database/TableCreator.html]

TableCreator


JavaScript is disabled on your browser.


- Overview
- Package
- Class
- Use
- Tree
- Deprecated
- Index
- Help

- Prev Class
- Next Class

- Frames
- No Frames

- All Classes

- Summary:
- Nested |
- Field |
- Constr |
- Method

- Detail:
- Field |
- Constr |
- Method


uk.ac.standrews.utils.main.database

## Class TableCreator

- java.lang.Object
- - uk.ac.standrews.utils.main.database.Manager
  - - uk.ac.standrews.utils.main.database.DbManager
    - - uk.ac.standrews.utils.main.database.TableManager
      - - uk.ac.standrews.utils.main.database.TableCreator

- All Implemented Interfaces:
  :   java.io.Serializable

  ---

    

  ```
  public class TableCreator
  extends TableManager
  ```

  Class to create a table object in the database. The table data (name etc.) is
  taken from a `Table` object.

  Version:
  :   10 Jun 2010

  Author:
  :   Luna De Ferrari luna.deferrari-at-ed.ac.uk

  See Also:
  :   Serialized Form

- - ### Field Summary

    - ### Fields inherited from class uk.ac.standrews.utils.main.database.DbManager

      `m_dbWriter`
  - ### Constructor Summary

    Constructors

    Constructor and Description

    `TableCreator(Table table)`
  - ### Method Summary

    Methods

    | Modifier and Type | Method and Description |
    | --- | --- |
    | `boolean` | `createTable()` Creates the database table for this `Table` object |
    | `void` | `dropCreateTable()` Drops and creates table corresponding to this Table object. |
    | `boolean` | `dropTable()` Drops the database table for this Table object. |
    | `java.lang.String` | `getSqlToCreateTable()` Get the SQL statement to create the table |

    - ### Methods inherited from class uk.ac.standrews.utils.main.database.TableManager

      `getTable, getTableName`
    - ### Methods inherited from class uk.ac.standrews.utils.main.database.DbManager

      `closeConnection, connectionIsValid, getDatabaseName, getDbConnection, getDbCreator, getDbReader, getDbWriter, refreshConnection, runAnalyzeTable`
    - ### Methods inherited from class java.lang.Object

      `equals, getClass, hashCode, notify, notifyAll, toString, wait, wait, wait`

- - ### Constructor Detail


    - #### TableCreator

      ```
      public TableCreator(Table table)
      ```
  - ### Method Detail


    - #### createTable

      ```
      public boolean createTable()
      ```

      Creates the database table for this `Table` object

      Returns:
      :   true if the table was created, false if the table could not be
          created or already exists in database


    - #### dropCreateTable

      ```
      public void dropCreateTable()
      ```

      Drops and creates table corresponding to this Table object. Beware! risk
      of data loss. Better to rename the table and relaunch than dropping it.

      Throws:
      :   `java.sql.SQLException`


    - #### dropTable

      ```
      public boolean dropTable()
      ```

      Drops the database table for this Table object. Beware! risk of data
      loss. Better to rename the old table and relaunch than to drop the table.

      Returns:
      :   true if the table does not exist in the database anymore


    - #### getSqlToCreateTable

      ```
      public java.lang.String getSqlToCreateTable()
      ```

      Get the SQL statement to create the table

      Returns:
      :   the create table statement, including all the fields definitions
          and primary keys


- Overview
- Package
- Class
- Use
- Tree
- Deprecated
- Index
- Help

- Prev Class
- Next Class

- Frames
- No Frames

- All Classes

- Summary:
- Nested |
- Field |
- Constr |
- Method

- Detail:
- Field |
- Constr |
- Method
